# SEaRCH™ expert panel process: streamlining the link between evidence and practice

**DOI:** 10.1186/s13104-015-1802-8

**Published:** 2016-01-07

**Authors:** Ian Coulter, Pamela Elfenbaum, Shamini Jain, Wayne Jonas

**Affiliations:** RAND Corporation, 1776 Main Street, Santa Monica, CA 90401 USA; Samueli Institute, 1737 King Street, Suite 600, Alexandria, VA 22314 USA

**Keywords:** Clinical expert panel, Research expert panel, Policy expert panel, Patient expert panel, Subject matter experts, Methodology, Appropriateness, Scientific evaluation and review of claims in health care

## Abstract

**Background:**

With rising health care costs and the diversity of scientific and clinical information available to health care providers it is essential to have methodologies that synthesize and distill the quality of information and make it practical to clinicians, patients and policy makers. Too often research synthesis results in the statement that “more and better research is needed” or the conclusions are slanted toward the biases of one type of stakeholder. Such conclusions are discouraging to clinicians and patients who need better guidance on the decisions they make every day.

**Method:**

Expert panels are one method for offering valuable insight into the scientific evidence and what experts believe about its application to a given clinical situation. However, with improper management their conclusions can end up being biased or even wrong. There are several types of expert panels, but two that have been extensively involved in bringing evidence to bear on clinical practice are *consensus panels*, and *appropriateness panels*. These types of panels are utilized by organizations such as the National Institutes of Health, the Institute of Medicine, RAND, and other organizations to provide clinical guidance. However, there is a need for a more cost effective and efficient approach in conducting these panels. In this paper we describe both types of expert panels and ways to adapt those models to form part of Samueli Institute’s Scientific Evaluation and Research of Claims in Health Care (SEaRCH™) program.

**Discussion:**

Expert Panels provide evidence-based information to guide research, practice and health care decision making. The panel process used in SEaRCH seeks to customize, synthesize and streamline these methods. By making the process transparent the panel process informs decisions about clinical appropriateness and research agenda decisions.

## Background

When attempting to develop evidence-based medicine, health professions face several challenges in trying to base their practices on evidence. One source of information is scientific evidence derived from research studies. However, since a single study is seldom definitive, a systematic review of the numerous studies published in the literature is preferable. Systematic reviews of the literature provide a method of assessing the quality of each individual study [1, 2], as well as assessing the overall level of evidence based on the entire published literature for a particular intervention in a given population [2–5]. In a separate article in this series of articles we describe a rapid and reliable way of getting sound evidence through systematic reviews called the Rapid Evidence Assessment of the Literature (REAL^©^) [6]. However, while clinical research, systematic reviews and their publications are necessary, they are not sufficient to ensure that the relevant evidence has been properly assembled or that it can influence practice and clinical decision making. This may occur because of the paucity of studies available, because of the poor quality of the studies, because of the restricted nature of the populations in clinical studies or because of the impossibility of doing the studies for either ethical issues or methodological or resource reasons [6, 7]. The result is that much of health care occurs in a clinical space in which the evidence is indeterminate or uncertain. When good evidence does not exist to guide practice, clinicians must base their approach on their own clinical intuition, on what they were taught in their training or on what the experts recommend. However, such approaches lack transparency and rigor and are often fraught with error. Coulter has termed this “therapeutic anarchy” where in effect each clinician does his/her own thing or the market drives what is done rather than patient needs and preferences [7, 8]. The situation is even worse for patients who may not even have the basic knowledge or experience on how to interpret evidence but are also asked to make decisions about treatment choices and preferences.

## Expert panels

One approach that has emerged to overcome this problem has been the use of expert panels (EP). While in systematic reviews the opinion of experts is considered at the bottom of the evidence hierarchy, in expert panels, which combine evidence and clinical acumen, the opinions are made transparent and subjected to critical appraisal. This overcomes the major objection to the opinion of experts vs. systematic reviews and has the additional value of allowing the introduction of information closer to clinical practice and a better interface with research evidence.

Samueli Institute uses the following expert panels: a Clinical Expert Panel (CEP), and a Research Expert Panel (REP), with others designed for policy (PoEP) and patient expert panel (PaEP) decisions, in development. These types of panels form a part of the Scientific Evaluation and Review of Claims in Health Care (SEaRCH™) process. [6] They draw on the best in existing models of expert panels, but differ in that they are designed to complement the evidence process in the SEaRCH program to better clarify both research agenda needs and facilitate practice decisions. They also differ from other EPs in that the process for doing them is structured and streamlined to ensure they can be conducted rapidly and in an objective and cost effective manner. Furthermore, they can be customized in response to a client’s needs.

Samueli Institute’s EP processes have drawn from three methods developed over time to deal with the application of evidence to practice. These are the National Institutes of Health Consensus Development Conference (NIH CDC), the Institute of Medicine (IOM) report process and the RAND Expert Panel process. Because we draw on these three methods they will be described briefly in what follows.

## NIH consensus panels

The National Institute of Health (NIH) consensus development conference method for resolving evidence judgment issues was begun in 1977 by NIH as a method by which the scientific community could bring relevant research to bear on the quality of health care [8–10]. The purpose of the NIH Consensus Development Conference (CDC) is to bring clinical practice more in line with research and so improve the quality of care. “To achieve this, the focus is on the scientific community bringing to the attention of the health professions the results of research and the impact on the quality of care.” [8, 9] Given its purpose the membership of the NIH panels favors research experts in both the clinical and related aspects of the topic.

NIH panels focused largely on questions of efficacy and safety [10], and are intended to resolve scientific issues (controversies) [11]. However, the issues chosen have to be resolvable by evidence. There is testimony from experts, audience participation and the end product of the panel is consensual statements (i.e. 100 % agreement). A panel of experts is chosen and reviews the evidence for or against a procedure. Recently, panels are provided with a systematic literature review and the original literature. Over 2 days panelists also hear testimony from experts. These hearings are open to audience participation who may also comment. At the end of this process the panel is then cloistered until the final consensual recommendations are completed. These are then issued publicly. The topics chosen can either be disease based (e.g. breast cancer) or procedure based (e.g. mammography). The focus is on the state of the science rather than the state of current practice. Commentators have described the NIH process as a combination of three models: the judicial process using testimony and a jury; a scientific meeting where research evidence is presented; and the town meeting where interested citizens can make comments [9, 10]. Ultimately, however, the recommendations are made by a select few with scientific expertise and made behind closed doors. Consensus panels may also comment on the state of the science and make recommendations about both present and future research needs.

There are, however, several challenges which limit the usefulness of the NIH Consensus Development Panels. If the focus is only on those issues that can be resolved by scientific evidence, it is necessarily confined to those issues with scientific support. Unfortunately, within the field of health care, many of the most problematic issues cannot be resolved by evidence alone. In such a situation the NIH CDC is forced to report simply on the state of the science. In addition, the judgment processes used for the final conclusions are also done behind closed doors and so are not completely transparent.

The NIH panel places a premium on research findings, and gives less weight to clinical experience or acumen (although in the testimony phase it may hear this). This undermines the credibility of the NIH panels with the very persons it seeks to influence—practitioners and patients. Additionally, the panels favor largely scientific (not clinical experts) in both the clinical and related aspects of the topic [8, 9]. Related to this, they focus on efficacy and not effectiveness and on the state-of-art practices and not usual practice. This makes their guidelines less useful to practitioners. This panel is an expensive process which means it cannot be repeated very often. Where technology is transforming practice rapidly, the findings can become obsolete very quickly. Consensus statements are considered by NIH to be historical documents after 5 years and they do not recommend that decisions be based on these statements after that time. Unfortunately, updates to these statements are rarely conducted, due to the costs of conducting the comprehensive systematic reviews and the rarity of gathering an NIH panel. This is limiting in areas of health care that are changing rapidly, where answers about particular clinical procedures are needed expediently, and where it is recognized that evidence-based clinical decision-making needs to be informed via timely updates and re-assessment of the literature [13]. Research by RAND has shown that the impact of the NIH panels on care is not strong in changing physician behavior [10–12].

Another limiting factor of NIH panels is that they are consensus-based, and while minority reports are possible, participants are strongly encouraged to agree on assessments and recommendations. Such consensus panels may be more prone to bias from dominant panel members [11]. The recommendations include only those for which there is consensus, and the panels are cloistered until such consensus is met [12]. This often means that the result is the “lowest common denominator”. The evidence for the clinical impact of the NIH panels on changing behavior of health care providers has not been strong. The emphasis on efficacy rather than effectiveness and not on the state of the current practice has limited the relevance of the panel findings for a broader health care audience.

In situations where the evidence is insufficient to derive consensus statements or as part of the consensus panel for clinical practice, a state of the science special panel can be created. This panel is also further limited by the relative lack of transparency and specificity related to gap assessment and research recommendations. Little systematic information is given on how group decisions are made regarding literature gap assessment, including the relevance of a particular gap for clinical practice or policy, or the types of research designs best suited to address specific gaps. Finally, as noted by the Institute of Medicine (IOM), many expert panels do not make a clear distinction between the quality of evidence vs. strength of recommendations. While the NIH State-of-the-Science Panels are ostensibly run when there is a perception that the evidence base is “weaker”, the methods used in assessing the quality of evidence may vary and strength of recommendations often varies. Recommendations from these “State-of- the-Science” panels are not presented based on the relevance of a particular gap in terms of research, practice, or policy.

## Institute of medicine reports

The Institute of Medicine (IOM) also produces reports that often summarize evidence and make recommendations about practice and research. While their methods can vary, the basic approach is similar to the NIH CDC in that they pick scientific experts, receive outside input and sequester the participants and seek consensus whenever possible. Unlike the NIH CDC, the panels usually meet several times and have more time for discussion and analysis. In addition, the reports are longer and more thoroughly developed. However, despite a recent IOM report on criteria for systematic reviews and guidelines, each panel does not yet always conduct or rely on systematic reviews and their processes are sometimes even less transparent than those of NIH [12, 13]. Their impact on clinical practice also varies. While IOM works hard to reduce clear conflict of interest of panel members, they do not structure the conduct of any SRs done and the experts in a way to prevent bias during the assessment and consensus process. Thus, the bias of panel members with more dominant voices can influence the outcome.

## RAND expert panels for appropriateness of care

The RAND method has been extensively described elsewhere in the literature. One of its essential features vis-a vis the NIH or IOM approaches is that the RAND approach is oriented less to scientific experts and more to clinical experts [8, 9]. In addition, the RAND process has carefully evaluated the optimum process for creating diverse and multi-stakeholder input, allowing for bias reduction and wider audience relevance.

Coulter [13, 14] has described the RAND panel in previous publications and this will be drawn on here [14, 15]. The process has a specific way of managing the panel to address some of the limitations of those previously described. In a RAND panel nine experts are chosen. The members reflect a spectrum of clinicians and academics, so that any given specialty does not dominate the panel (five academically based and four practicing clinicians). In addition, the RAND expert panel evaluation of the evidence process departs from that of the NIH CDC and IOM. The main differences between these and the RAND panel process are described below.

First, in a RAND panel an extensive review of the literature is conducted and a systematic review written (a meta-analysis if this is possible, but a synthesis if it is not) [23]. In the RAND clinical appropriateness panel process, research staff (with input from clinical expertise) then creates a set of possible clinical indications to which the evidence might be applied. These indications categorize patients in ways that they usually present to the clinic. This includes such things as their symptoms, past medical history, the results of previous diagnostic tests and patient preferences. “The objective is to create lists that are detailed, comprehensive, and manageable. They must be detailed enough so that the patients covered by the category are relatively homogeneous so that the procedure would be equally appropriate for all of them. To be comprehensive they must include all the indications for doing the procedure that occur in practice. However, they must be short enough that the panelists can complete them within a reasonable space of time.” [8, 9].

After these lists are compiled, the process uses a modified Delphi method where the indications created are sent to the nine panelists along with the literature synthesis. The panelists then independently rate the appropriateness of the procedure based on the evidence from the literature review and their clinical experience. The ratings for appropriateness are from 1 to 9, with 1 representing extremely inappropriate and 9 extremely appropriate. Appropriate care is defined as when “the expected health benefit to the patient (relief of symptoms, improved functional capacity, reduction of anxiety, etc.) exceed expected health risks (pain, discomfort, time, cost, etc.) by a sufficiently wide margin that the procedure is worth doing” [14, 15]. The panelists are instructed to evaluate the risks and benefits based on commonly *accepted best clinical practice* for the year in which the panel is conducted. Considering an average group of patients with each listed indication, presenting to an average practitioner in the US, the ratings should reflect the panelist’s own best clinical or expert judgment. In this way the judgments are a combination of evidence from the literature and clinical acumen or experience with realistic variations as they often happen in the clinic. This forms a bridge from evidence to practice in a more realistic way than simple evidence summaries do, but with a more systematic and balanced process than individual clinical opinion.

These ratings are quantitatively summarized by the research staff. Then, in a second round of ratings the panelists are brought together in a face-to-face meeting. Each panelist is shown his/her rating for each indication, and the distribution of the ratings of the panel as a whole is presented for each indication. Individual panelists must reconsider their judgment and although they are not forced to defend it in most cases, where the individual does differ from the group, he/she will usually do so or at least explain the logic or evidence for that position. Following the discussion, the panelists re-rate the appropriateness of the procedure again. From the second rating, it is possible to determine the degrees of agreement in the panel, and to calculate the average median ratings, and the average dispersion measures for the procedures. Consensus is not required. However, in most instances the dispersion decreases during the second rating as the panelists come closer to a consensus. Once the work of the expert panel is completed, the team then compiles a set of indications for performing a procedure based on the evidence and their clinical experience, which can then be used to compare to actual practice. This allows researchers to calculate a rate of appropriate/inappropriate care present in practice.

In the RAND panels, consensus is not required but its degree is measured. RAND has utilized two approaches to measure consensus. In the first there is consensus if all raters’ responses fall within one of the three point ranges of the scale (i.e. 1, 2, 3; 4, 5, 6; 7, 8, 9). This would mean all the raters agreed that the procedure should not be performed (1, 2, 3); they agreed that it was questionable or uncertain (4, 5, 6); or, they agreed that it should be performed (7, 8, 9). The second method is to define agreement if all the ratings fell within any 3 point range. Furthermore, agreement can be determined using both methods but by rejecting one extreme high or low rating. Similarly, disagreement can be calculated using two methods; if at least one rater chose a 1 and one chose a 9; or if at least one rater fell in the lowest three point region and at least one in the highest. As with disagreement, the extreme ratings can be discarded. A procedure can be judged inappropriate if its median rating is in the range 1–3, and without disagreement; uncertain if the median rating is in the range 4–6, and appropriate if it is 7–9, without disagreement. Finally, the outcome can also be that the panelists disagreed on the proper rating (they were indeterminate) [13–16].

A unique feature of the RAND appropriateness panel is the amount of research that has been done on its reliability and validity, making the RAND panel process, unlike other expert panel processes, truly evidenced-based. For validity of the RAND appropriateness panel, studies have examined the relationship between ratings and the literature [6], face and content validity [16, 17] and construct validity [18, 19]. Studies have looked at test–retest reliability [20], compared panels occurring in different countries on the same procedures [21], compared panels occurring at different times [20], and investigated the impact of panel membership on the judgments of appropriateness [17]. These studies show that, when these steps are applied, extreme variation across the panels does not occur. The first formal test of reproducibility of the RAND panels [20] tested the reliability of three parallel panels for two procedures, hysterectomy and coronary revascularization, conducted within the same time frame. Comparing the reproducibility of the panels with what physicians do daily, the study concluded that the RAND method is much less variable than physicians making independent decisions. Coulter et al. [17] have compared the panel ratings of a multidisciplinary panel versus an all specialty panel for spinal manipulation for low back pain and shown that those who use this procedure are more likely to rate it as appropriate for more conditions than those who do not [7, 17–22].

## SEaRCH™ Expert Panels

The SEaRCH Expert Panel Process draws heavily on the work done at NIH, IOM and RAND. The process integrates the most reliable and useful aspects of these panels. The desire to create a streamlined approach was driven by the need to reduce cost and create more efficient and transparent expert panels.

One example of streamlining is to have expert panelists utilize the online technology database to enter data related to appropriateness ratings. Another is to hold meetings via teleconference, which allows for reduced costs as well as the inclusion of key expert panelists that otherwise may not be able to attend an in-person meeting. This also allows for the overall panel process to happen more expediently and to be more inclusive of the right experts for balance. In addition, it allows for a greater variety of panel types to be developed to serve different purposes, such as panels focused on policy and patient preferences.

Currently, the SEaRCH Expert Panel Process (SEPP) uses expert panels for two primary purposes –clinical appropriateness and research agendas. A Clinical Expert Panel (CEP) is used to determine when care is appropriate or inappropriate combining both evidence and clinical acumen, similar to the RAND process. A Research Expert Panel (REP) is designed to examine the state-of-science and to identify and prioritize gaps in the scientific evidence vis-à-vis practice. Both the CEP and REP are used particularly in areas where the evidence is either lacking in quality or insufficient to determine appropriate care using usual consensus or guideline processes. While the two expert panels differ, the processes they use are similar and will be described jointly below.

## SEaRCH Expert Panel Process (SEPP)

**Fig. 1:**
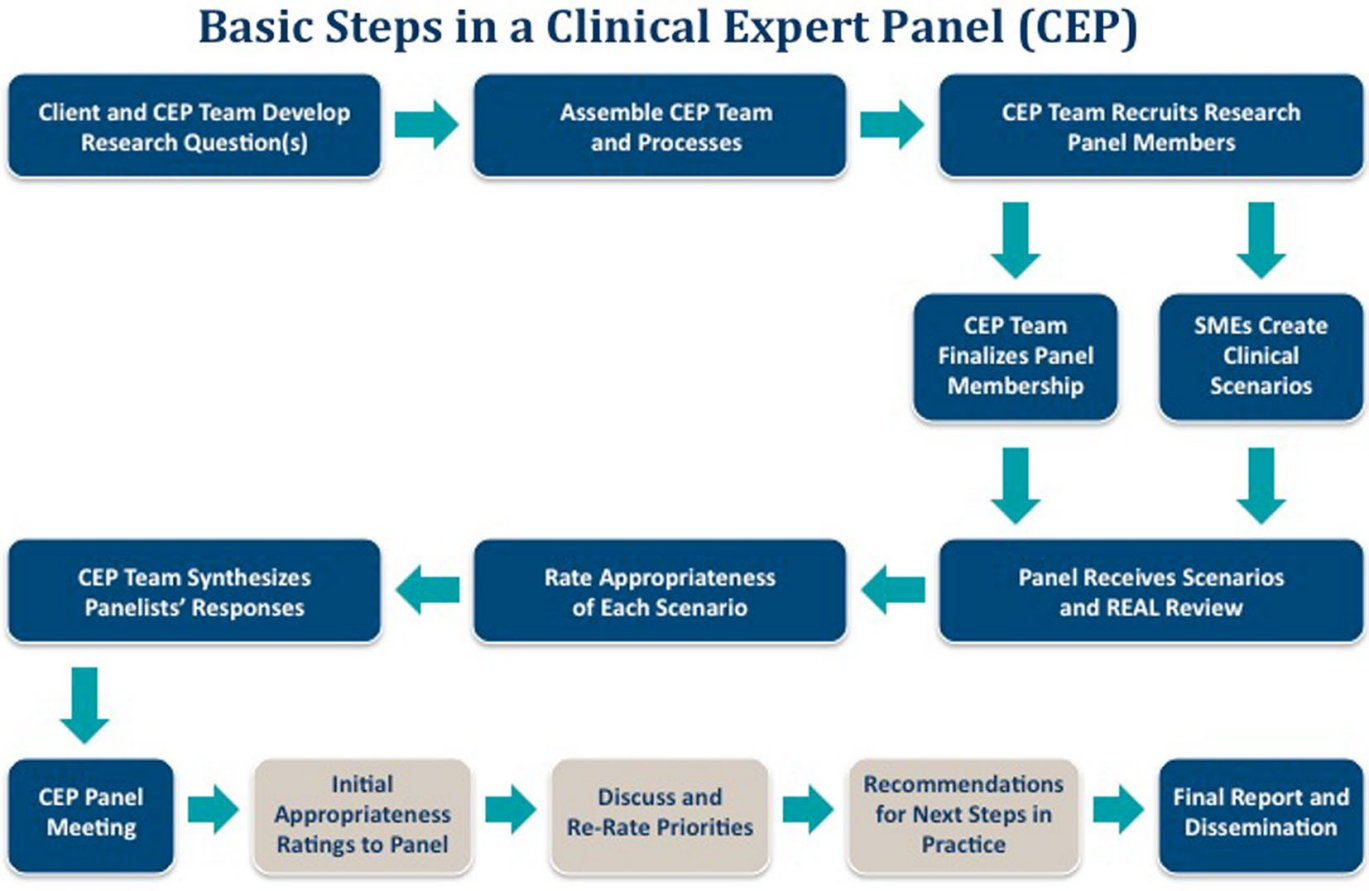
Basic steps in a clinical expert panel (CEP)

A request for a SEaRCH Expert Panel Process (SEPP) typically comes from an outside person or group who needs a balanced and objective way to make research or clinical recommendations on a particular topic. The requestor often seeks to obtain an evidence-based, expert judgment to help determine the appropriateness for clinical use of, or develop the research needs for, a product, practice, program or policy. In the case of a clinical expert panel (CEP), the requestor seeks recommendations for an intervention within a given setting or recommendations around a particular treatment approach in various settings. In the case of a research expert panel (REP), the requestor seeks specific research recommendations based on current gaps in the evidence and may include an assessment of available resources and readiness to conduct such research. The following describes the steps in the SEaRCH Expert Panel Process (SEPP). Figure 1 provides a flow chart of the CEP process.

The first step of the SEPP is for an Expert Panel Manager to meet with the client to create and refine the question to be answered. This meeting also examines the focus of the other integrated SEaRCH components such as the REAL (Rapid Evidence Assessment of the Literature) which is a streamlined, reliable, systematic review process; [22, 23] and the Claim Assessment Profile (CAP) [23, 24], which provides a detailed descriptive evaluation of the intervention (product, practice, program or policy) and claim (efficacy, effectiveness, efficiency, relevance, cost and outcome). The CAP and REAL processes involved in SEaRCH are described in the companion pieces in this issue. Next, a balanced team is created for the successful conduct of the panel. The selection of panel members is key for obtaining valid judgments from a panel. Panelists are always selected to ensure no conflict of interest in the area. They are also selected to provide a diversity of experience and knowledge within the panel. The SEPP process uses specific criteria to select the most qualified panel members.

As stated earlier, one of the benefits of the SEaRCH model is the communication that occurs between the description, evidence and judgment components (the CAP, REAL and SEPP, respectively) needed to answer important health information questions. For example, using the REAL process for informing the Expert Panels provides a systematized assessment of research quantity and quality. For the clinical expert panel, specific SMEs develop the clinical scenarios that will be rated by the panelists. Once the EP team and panel members have been established and the clinical scenarios created, the actual panel process begins.

Each panelist integrates information from the REAL and/or CAP evidence with their own clinical judgment for each scenario. They rate the appropriateness of use of the intervention for each scenario and enter their ratings into an online database. The use of the database allows for less error, more opportunity for statistical review and a much faster turnaround of results for phase II.

The second phase of the CEP consists of all nine panelists meeting face to face, either in person or through virtual means to review their clinical appropriateness rating scores and discuss them among the group. After the panel discussion, panelists are asked to re-rate the scenarios using the online database. The computerized program can be used to tabulate the new re-rated scenarios.

## Conducting research expert panels

**Fig. 2:**
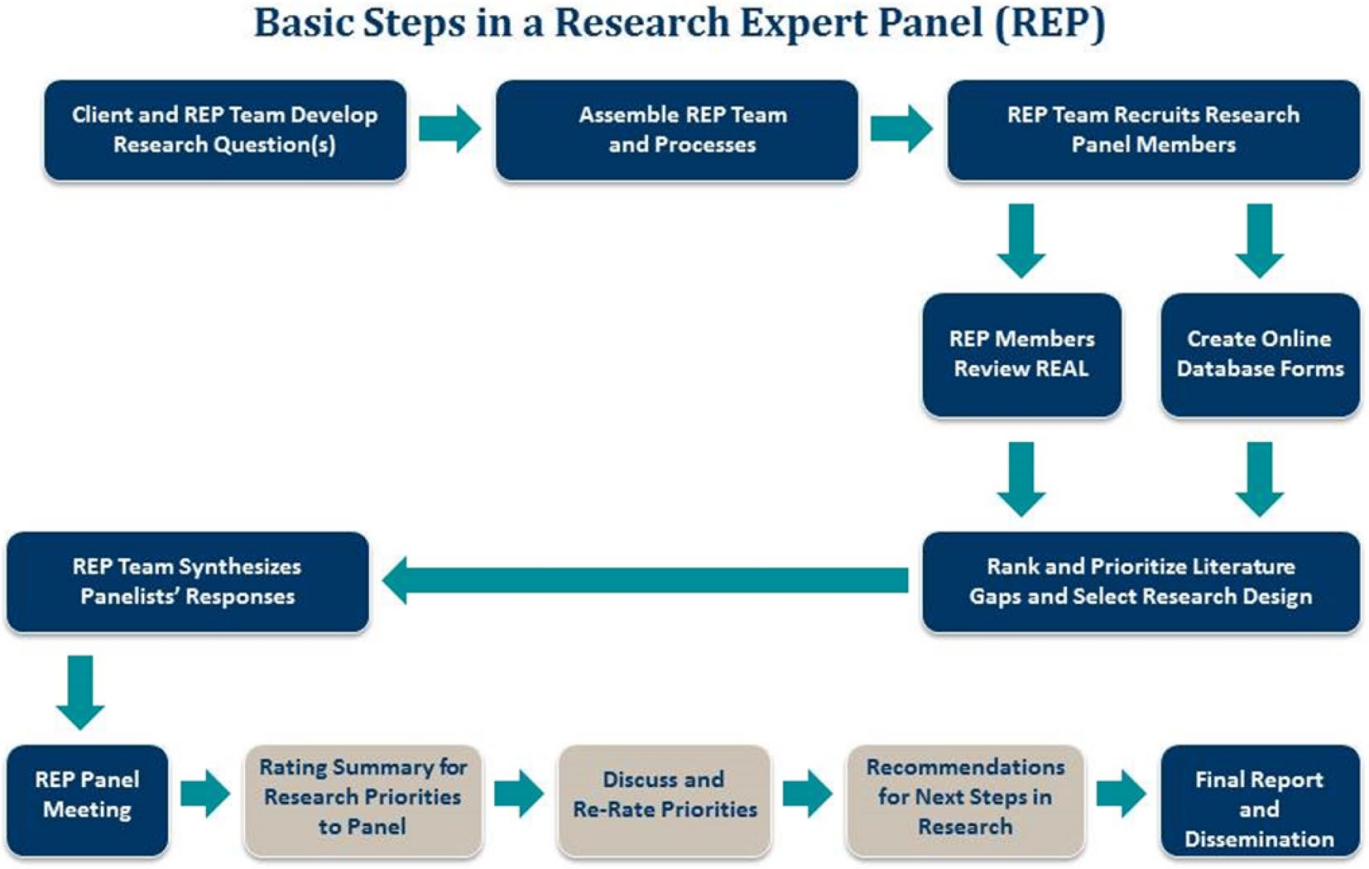
Basic steps in a research expert panel (REP)

As the flow chart in Fig. 2 demonstrates, the initial steps in the research panel are similar for the research and clinical expert panels. The following methodological processes focus on the specifics of conducting the Research Expert Panel.

Similar to the Clinical EP, the Research EP is a two-phased process. However, in this case, initial ratings entered in the online technology database are not based on appropriateness of clinical care, but rather on elucidating and clarifying research gaps, as well as identifying research designs to address the most relevant gaps.

Similar to the Clinical EP, the Research EP member integrates his/her knowledge and expertise with the available evidence from the REAL and/or CAP, and then enters this information into a computerized database. The initial ratings provided by the research expert panelists help to systematize and make transparent specific recommendations on research directions in particular gap areas. In the case where research recommendations are being made specifically for an institution that wishes to carry out a research project, tailored recommendations also are based on the available resources for that institution (when explicated through the CAP). The process for convening the Research Expert Panel in Phase II is similar to the Clinical Expert Panel.

The goal of the research expert panel meeting, however, is for the panelists to discuss areas where they may disagree on gaps and next research priorities. As with the clinical expert panel, after the panel discussion, panelists are then asked to re-rate the research form. Panelists input their ratings in the database which tabulates the scores.

The last phase of the process is to summarize and deliver the panel recommendations in a report with quantified ratings. Recommendations and specific content of each summary report will vary depending on the type of panel (research vs. clinical appropriateness) and the question(s) that the client is seeking to answer. For these panels, the summary includes an in depth description of the EP methodology and recommendations based upon the findings.

## Panel variations: making panels more patient centered

It is important to note that this panel model can be modified to address questions on multiple issues related to health care such as policy implementation and patient-centeredness. For example, a Policy Expert Panel (PoEP) is a derivative of the Research Expert Panel and focuses on making evidenced based policy judgments (payment, coverage, scope of practice) to direct implementation of a practice claim. In fact, this panel is often used to explore direct implementation issues even when a policy issue is not a factor.

One of the biggest challenges in all panel processes is that they have difficulty obtaining patient input early and continuously in the decision making. Usually, patient representatives are placed on advisory boards where input comes late in the decision making process. In addition, patients may feel intimidated or lost when on panels with scientists and clinical experts, especially if they are not trained or comfortable executing their role and using the panel methodology. The Samueli SEPP has patients and patient interest groups involved in every phase of the process,. It may even be deemed appropriate to convene a Patient-only Expert Panel (PoEP) to achieve a complete patient perspective. Patients can be incorporated into panels in various ratios such as equal (1:1:1 with scientists and clinicians) or weighted toward patients (2:1:1). In addition, use of the anonymous Delphi and virtual meeting processes can further empower patient input more deeply in judging either clinical or research relevance, making both methods more patient-centered. The REAL review process also trains all potential panel members (no matter their expertise) in how to use the results of a systematic review. Special training of the patient panel members and of panel moderators also enhances communication and input from the patient’s perspective [23, 25].

## Conclusions and discussion

There is an essential need for evidence-based information to guide research, practice and health care decision making. Expert panels contribute to evidence-based decision making in both research and practice by closing the gap between the usual evidence summaries and the needs of clinicians, policy makers, patients and researchers for using such evidence in daily decisions. There is a clear need for more transparent, systematized, and efficient processes for expert panels, especially in terms of providing recommendations. This process seeks to fill those gaps. As part of the SEaRCH program, Samueli Institute streamlined an expert panel process that was driven by the need for cost effective, efficient and transparent approaches to addressing health care needs. It is designed to expand diverse stakeholder input into the research judgment process. This allows for easier delivery of expert opinion and patient input for making decisions about research priorities and clinical appropriateness. This methodology is integrated with other SEaRCH components such as the Rapid Evidence Assessment of the Literature (REAL) and the Claims Assessment Profile (CAP). This methodology is still new and will need to be validated in the future. The use of information from these three evidence based components, will allow for more customized, expedient and evidence-based recommendations on therapeutic claims of interest.

The expert panel process is the final step in the integrated SEaRCH process described in this series of articles. It improves the link between research evidence, research agendas, real world practice decisions and patient needs and preferences. Together these integrated strategies allow development of true evidence-based health care.

## Abbreviations

CAP: claims assessment profile
CEP: clinical expert panel
EP: expert panel
IOM: Institute of Medicine
NIH CDC: National Institutes of Health Consensus Development Conference
PaEP: patient expert panel
PoEP: policy expert Panel
REAL^©^: Rapid Evidence Assessment of the Literature
REP: research expert panel
SEaRCH™: scientific evaluation and review of claims in healing
SEPP: SEaRCH™ expert panel process
SME: subject matter expert
SR: systematic review
